# Pharmacist-driven antimicrobial stewardship: evolving roles in clinical practice

**DOI:** 10.1186/s40780-026-00576-0

**Published:** 2026-04-25

**Authors:** Naoto Okada

**Affiliations:** Pharmacy Department, Yamaguchi University Hospital, 1-1-1 Minamikogushi, Ube, Yamaguchi 755-8505 Japan

**Keywords:** Antimicrobial resistance, Antimicrobial stewardship, Pharmacist, Prospective audit and feedback

## Abstract

Antimicrobial resistance (AMR) poses a major global public health threat. The effective implementation of antimicrobial stewardship (AS) is essential for optimizing antimicrobial use and improving clinical outcomes. In this context, pharmacists play a central role by managing pharmacotherapy across multiple clinical practice domains. This narrative review summarizes evidence on pharmacist-driven AS, focusing on three key areas: (i) the facilitation of proper antimicrobial use, (ii) the management of antimicrobial-related adverse events, and (iii) support for evidence-based infection control and prevention. Prospective audit and feedback, surveillance of antimicrobial consumption and outcomes, diagnostic stewardship, and educational interventions represent the core strategies through which pharmacists optimize antimicrobial use. Additionally, pharmacists can enhance treatment safety by conducting structured risk assessments and monitoring drug-specific toxicities. Pharmacists further support infection control practices through disinfectant management, outbreak response, and post-exposure prophylaxis. Finally, the integration of real-world data and structured workforce development programs is essential for sustaining and advancing pharmacist-driven AS. This review highlights the role of pharmacists as leaders in the implementation and continuous refinement of AS tailored to institutional and regional healthcare needs.

## Background

Antimicrobial resistance (AMR) represents a global public health challenge for both healthcare systems and patient outcomes [1]. While multiple factors contribute to the emergence and spread of AMR, the quantity and quality of antibiotic use are key modifiable drivers, making antimicrobial stewardship (AS) a central strategy in healthcare [2, 3]. By minimizing unnecessary exposure to broad-spectrum antibiotics, AS reduces selection pressure, thereby mitigating the emergence and dissemination of resistant organisms and contributing to improved patient outcomes [4–6]. Accordingly, the effective implementation of AS in routine care is essential to combat AMR.

In hospital settings, AS is typically implemented through collaboration between attending physicians and an AS team composed of infectious diseases specialists [7]. As integral members of this team, pharmacists contribute to AS by providing specialized expertise in antimicrobial pharmacotherapy, including facilitating the proper use of antimicrobial agents, managing antimicrobial-related adverse events, and supporting evidence-based infection control and prevention. These contributions underscore the value of integrating pharmacists into core AS activities.

This narrative review provides an overview of the implementation of pharmacist-driven AS, in which pharmacists proactively initiate, design, and implement interventions, as well as the roles that pharmacists play in AS.

## Role of pharmacists in the facilitation of proper antimicrobial use

### Prospective audit and feedback

**Fig. 1:**
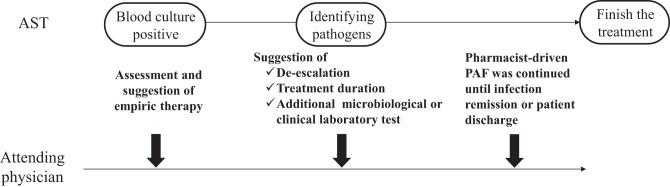
Framework for pharmacist-driven PAF in patients with bloodstream infections. Abbreviations: AST, antimicrobial stewardship team; PAF, prospective audit and feedback

**Fig. 2:**
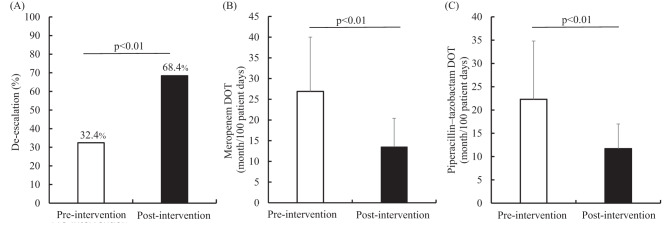
Outcomes of implementing the pharmacist-driven PAF framework. Changes in de-escalation rates before and after intervention (**A**). Change in days of therapy with meropenem (**B**) and piperacillin–tazobactam (**C**) before and after the intervention. Abbreviations: DOT, days of therapy

One of the core activities of AS is prospective audit and feedback (PAF). This involves continuous review and recommendations aimed at optimizing antimicrobial treatment for prescribers [8–10]. We previously developed and implemented a pharmacist-driven PAF framework for patients with bloodstream infections (Fig. 1) [11]. In this framework, pharmacists screened electronic medical records to identify patients with positive blood cultures and reviewed corresponding microbiological reports. Antimicrobial therapy for bloodstream infections was prospectively audited, focusing on the appropriateness of agent selection, dosing, treatment duration, the need for de-escalation, and the adequacy of culture collection. When potential issues were identified, the pharmacists discussed optimization strategies with the attending physicians, including pathogen-directed therapy, dose adjustment, evaluation of possible contamination, and the need for additional microbiological testing. Following its implementation, we observed increased rates of de-escalation and a reduction in therapy days with carbapenems and piperacillin–tazobactam (Fig. 2).

In a study focusing on extended-spectrum beta-lactamase-producing *Escherichia coli* bacteremia, Yamada et al. demonstrated that the introduction of PAF markedly increased de-escalation from broad-spectrum agents to narrower-spectrum agents such as cefmetazole in selected cases based on antimicrobial susceptibility and clinical severity, while carbapenems remained necessary for patients with severe infection or high-risk features [12]. Nakano et al. evaluated pharmacist-driven interventions in a hospital without an infectious disease specialist and reported significant reductions in the days of therapy for carbapenem, with a significant decrease in 30-day mortality among patients with severe bacteremia [13]. Similarly, Sawada et al. showed that pharmacist-driven interventions in hospitals without infectious disease physicians were associated with a reduced length of hospital stay and antimicrobial expenditures [14]. These findings indicate that pharmacist-driven PAF in the management of bloodstream infections can reduce broad-spectrum antimicrobial exposure, improve the quality of care, and contribute to improved patient outcomes, regardless of the institutional size or the availability of infectious disease specialists. Pharmacists play a key role in ensuring the feasibility and safety of stewardship recommendations through their specialized expertise. Although approaches for advancing AS vary according to institutional staffing levels and organizational structures, establishing systems that enable pharmacists to consistently provide actionable optimization strategies is essential for the promotion of AS.

### Surveillance

Surveillance through the visualization of antimicrobial use and the impact of AS activities is also a key component in ensuring the proper use of antimicrobial agents [15]. Monitoring antimicrobial consumption enables the early detection of shifts in prescription patterns and supports the prioritization of interventions, as well as the optimization of human resources. These surveillance activities involve monitoring process indicators, such as antimicrobial use metrics, intervention frequency, and recommendation acceptance rates, as well as outcome indicators, including the incidence of resistant organisms and mortality [16, 17]. Recently, novel metrics for evaluating antimicrobial use have been developed, and their clinical utility is actively being evaluated [18]. Pharmacists are responsible not only for conducting and interpreting surveillance but also for using observed changes to refine interventions and educational strategies. Furthermore, through regional collaborative frameworks, pharmacists contribute to the expansion of AS activities beyond individual institutions by promoting cross-institutional surveillance efforts [19].

### Diagnostic stewardship

The appropriate utilization of microbiological and clinical laboratory information is essential for optimizing antimicrobial therapies. Diagnostic stewardship, which involves the collection of cultures, accurate interpretation of identification and susceptibility results, and timely integration of these findings into clinical decision-making, represents a key component for supporting AS [20]. In addition, antibiograms summarizing institutional or regional antimicrobial susceptibility patterns provide important guidance for empirical antimicrobial selection. The continuous evaluation of antibiogram data also allows for trends in the detection of resistant organisms to be observed within institutions [21]. Beyond optimizing pharmacotherapy, pharmacists serve as a bridge between laboratory data and clinical decision-making, thereby enhancing the precision of AS activities.

### Education

To ensure the proper use of antimicrobial agents at an institutional level, continuous educational and awareness activities for multidisciplinary healthcare professionals are essential [22]. Enhancing awareness through physician education is associated with a reduction in inappropriate antimicrobial prescribing [2]. Importantly, educational initiatives in AS are more effective when they integrate real-time feedback and dialogue into clinical practice. As central members of the AS team, pharmacists play a critical role in designing educational content and ensuring its sustained implementation.

Pharmacist engagement in facilitating the proper use of antimicrobial agents is a core component of AS. This requires a structured and integrative approach that combines various pharmacist-driven activities. Within this framework, pharmacists are key drivers of antimicrobial optimization in routine clinical practice.

## Role of pharmacists in managing antimicrobial-related adverse events

### Framework for adverse event management

While facilitating the proper use of antibiotics is central to AS, ensuring treatment safety represents an equally critical dimension of pharmacist-driven AS. Antimicrobial-associated adverse events are frequently encountered in clinical practice and may hinder the continuation of appropriate treatment [23]. Therefore, effective AS requires a systematic assessment of adverse event risk prior to treatment initiation, followed by ongoing monitoring and timely intervention during therapy. As key implementers of this framework, pharmacists are expected to contribute to optimizing operational processes and generating evidence to ensure the safety of antimicrobial treatment.

### Drug-specific risk stratification and monitoring

The risk of antimicrobial-associated adverse events is determined by interactions among multiple factors, including antimicrobial exposure intensity and duration, as well as patient-specific characteristics, such as renal function, comorbidities, and concomitant medications [24]. In addition, agents associated with relatively frequent toxicities require careful pre-treatment risk assessment and structured post-initiation monitoring. For example, anti-methicillin-resistant *Staphylococcus aureus* agents exhibit distinct adverse event profiles, necessitating individualized management strategies. For vancomycin (VCM), nephrotoxicity is the primary adverse effect [25]. Recently, area under the concentration–time curve (AUC)-guided therapeutic drug monitoring (TDM) has been reported to reduce nephrotoxicity compared to traditional trough-based monitoring strategies [26]. However, the concomitant use of piperacillin–tazobactam has been shown to increase the risk of nephrotoxicity independently of vancomycin exposure levels [27, 28]. Therefore, the prevention of vancomycin-induced nephrotoxicity requires not only optimizing drug exposure but also integrated management that incorporates concomitant medications and other patient-specific factors. Daptomycin (DAP) is frequently associated with elevated creatine phosphokinase levels and myopathy [29]. The concomitant use of statins has been identified as a risk factor for DAP-induced muscle toxicity, underscoring the importance of risk stratification based on co-administered medications [30, 31]. In addition, pharmacovigilance analyses have highlighted the signs of daptomycin-induced eosinophilic pneumonia, and discussions regarding its early recognition and management have been reported [32]. Linezolid is known to cause thrombocytopenia relatively frequently, with treatment duration having been identified as a major risk factor [33]. Moreover, risk stratification for teicoplanin-associated hepatotoxicity has been reported using composite indices, such as the bilirubin–albumin–FIB-4 score [34]. Thus, evidence in support of risk assessment and structured monitoring of antimicrobial-associated adverse events is essential for facilitating proper antimicrobial use while ensuring both treatment continuity and patient safety.

### Impact of AS on treatment safety

Multiple meta-analyses have demonstrated that AS implementation is associated with a reduction in the incidence of *Clostridioides difficile* infection (CDI) among hospitalized patients [35, 36]. In Japan, pharmacist-driven AS interventions have been reported to significantly reduce the incidence of CDI [37]. In addition, TDM-guided dose adjustments have been shown to contribute to the prevention of drug-specific adverse events [38]. These findings suggest that the implementation of AS activities in the prescribing and monitoring processes enhances the overall safety of antimicrobial therapy.

In infectious diseases, patient backgrounds tend to be highly heterogeneous, and the prevention and early detection of adverse events require coordinated intervention by the AS team. Pharmacists are expected to be involved in the management of antimicrobial-associated adverse events throughout the entire course of therapy.

## Role of pharmacists in supporting infection control and prevention

### Integration of AS and infection control

Infection control and prevention, along with AS, are fundamental strategies for reducing healthcare-associated infections and controlling outbreaks. Although AS and infection prevention and control are traditionally organized as separate programs, they function as complementary strategies in clinical practice [39]. Their integration occurs through coordinated interventions that link antimicrobial optimization with infection prevention efforts. Pharmacists integrate these practices through alignment of antimicrobial use optimization with infection prevention strategies, collaborative outbreak response, and implementation of prophylactic interventions. During the coronavirus disease 2019 (COVID-19) pandemic, the importance of pharmacists’ involvement in infection prevention and control was abundantly demonstrated [40]. Moreover, reports from community pharmacies during the COVID-19 era highlighted the significance of hygiene practices, including cleaning and disinfection, as well as public health education for residents [41]. Therefore, pharmacists play a key role in infection control and prevention.

### Environmental infection control

Effective infection control requires the comprehensive implementation of evidence-based measures, including standardized selection, management, and use of appropriate disinfectants, education on infection prevention practices, strict adherence to contact precautions, and structured monitoring with feedback mechanisms [42]. For example, in a reported outbreak of extended-spectrum beta-lactamase-producing *Escherichia coli*, successful containment was achieved through a combination of environmental sampling and active surveillance interventions, correction of cleaning procedures, and enhanced education for relevant personnel, including environmental services staff [43]. The effectiveness of disinfectants varies according to their active ingredients and formulation characteristics. Deviations in concentration, insufficient contact time, and variability in preparation procedures can compromise their efficacy [44]. By ensuring the appropriate selection, preparation, and supply of disinfectants, pharmacists play a key role in supporting environmental infection control.

### Post-exposure prophylaxis

Post-exposure prophylaxis (PEP) is an intervention aimed at preventing transmission within exposed populations. In the context of influenza, PEP has been shown to reduce secondary transmission [45, 46]. However, adherence to PEP among healthcare workers has been reported to be suboptimal [47]. Pharmacists can contribute to the successful implementation of PEP-based infection control strategies by providing medication support to ensure appropriate adherence.

Thus, pharmacists also play a pivotal role in infection control and prevention by using their pharmacological expertise to support evidence-based practices and operational implementation.

## Future perspectives for pharmacist-driven AS

### Toward the next stage of pharmacist-driven AS

To sustain and further expand these three domains of pharmacist-driven AS, new methodological and educational frameworks are required. While evidence from individual healthcare institutions remains important, evaluations based solely on single-center experiences are inherently limited by inter-institutional variability and patient population heterogeneity. Therefore, the next stage of AS development will need to involve the integration of data across multiple institutions to better identify shared challenges and continuously refine the evaluation metrics from a broader perspective.

### Utilizing real-world data

One promising approach to achieve this goal is to utilize real-world data (RWD). RWD comprise data on diagnoses and treatments generated during routine clinical practice [48]. Because RWD are not originally collected for research purposes, their volume, granularity, completeness, and longitudinal traceability can vary considerably depending on the data source. However, once their characteristics are understood and appropriately utilized, RWD can facilitate research on questions that are difficult to address through conventional clinical studies. In Japan, efforts have been made to generate new evidence by leveraging the comprehensive coverage and cross-institutional traceability of Diagnosis Procedure Combination data and administrative claims databases [49, 50]. In addition, infectious disease-specific RWD, such as the Japan Surveillance for Infection Prevention and Healthcare Epidemiology, provide the necessary infrastructure for the longitudinal assessment of antimicrobial consumption and susceptibility patterns, as well as benchmarks at regional and institutional levels [19]. Thus, the use of RWD is expected to facilitate the large-scale evaluation of antimicrobial use patterns. In fact, the integration of RWD represents a paradigm shift from intervention-based evaluation to system-level performance assessment.

### Workforce development beyond routine practice

To ensure consistent advances in pharmacist-driven AS, it will be necessary to move beyond practices that rely on individual experience and to systematically develop a workforce infrastructure, including training the next generation. In Japan, concerns have been raised regarding a decline in research activities among pharmacists specializing in infectious diseases [51]. Pharmacists must move beyond merely implementing AS in routine practice and cultivate the ability to identify unresolved challenges and translate research findings back into clinical applications. To support pharmacists aspiring to specialize in infectious diseases, structured educational opportunities and institutional frameworks that enable clear and sustainable career pathways will also need to be established.

## Conclusion

**Fig. 3:**
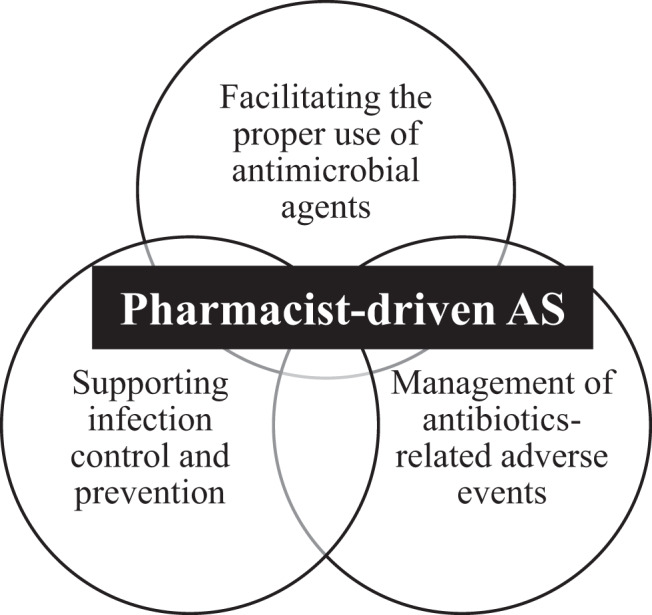
Key domains in which pharmacist-driven AS is needed. Abbreviations: PAF, prospective audit and feedback

This review provides a comprehensive overview of the roles that pharmacists play in AS implementation across several key clinical practice domains (Fig. 3). Importantly, the domains discussed herein should not be viewed as independent activities but as interconnected layers of pharmacist-driven AS. In the context of AMR, pharmacists are tasked with demonstrating leadership to establish and advance AS initiatives, particularly those tailored to the needs of individual institutions and regional healthcare systems.

## Data Availability

No datasets were generated or analysed during the current study.

## Abbreviations

AMR: Antimicrobial resistance
AS: Antimicrobial stewardship
PAF: Prospective audit and feedback
VCM: Vancomycin
AUC: Area under the concentration–time curve
TDM: Therapeutic drug monitoring
DAP: Daptomycin
CDI: *Clostridioides difficile* infection
COVID-19: Coronavirus disease 2019
PEP: Postexposure prophylaxis
RWD: Real-world data
